# A surge of AI-driven publications: the impact on health professionals and potential mitigating solutions

**DOI:** 10.3389/fpubh.2025.1680630

**Published:** 2025-09-24

**Authors:** Guglielmo Arzilli, Elisa Di Maggio, Luigi De Angelis, Francesco Baglivo, Elena Savoia, Gaetano Pierpaolo Privitera, Caterina Rizzo

**Affiliations:** ^1^Department of Translational Research and New Technologies in Medicine and Surgery, University of Pisa, Pisa, Italy; ^2^Hygiene Unit, Policlinico Foggia Hospital, Department of Medical and Surgical Sciences, University of Foggia, Foggia, Italy; ^3^Emergency Preparedness Research Evaluation and Practice Program (EPREP), Division of Policy Translation and Leadership Development, Harvard T.H. Chan School of Public Health, Boston, MA, United States; ^4^Department of Biostatistics, Harvard T.H. Chan School of Public Health, Boston, MA, United States

**Keywords:** artificial intelligence in health, scientific integrity, AI-generated publications, health professionals’ education, ethical guidelines in research

## Abstract

The rapid development of generative AI is reshaping scientific communication, particularly in medicine and public health. Since the release of ChatGPT in 2022, Large Language Models have become widely accessible, supporting manuscript editing, statistical analysis, and rapid evidence synthesis. However, this surge in AI-generated content raises concerns about the quality, reliability, and ethical implications of scientific publishing. Increased reliance on AI-driven authoring tools could exacerbate an “infodemic”—an overwhelming flood of potentially unreliable or misleading information. This risk is exacerbated by the prevailing “publish or perish” culture, which prioritizes publication volume over meaningful contributions. In addition, the proliferation of academic journals, especially those that charge high publication fees, deepens inequalities in global health research and limits access for low-income countries. Documented cases of fabricated articles and false authorship in predatory journals highlight how AI can be misused, threatening evidence-based medicine and influencing healthcare decisions. To address these challenges, regulatory frameworks, ethical guidelines, and widespread digital literacy training for researchers and health professionals are critical. A balanced approach—harnessing the efficiency of AI while safeguarding scientific integrity—is needed to prevent an AI-driven infodemic and ensure the equitable, high-quality dissemination of medical knowledge.

## Introduction

Ioannidis et al. (1) observed that thousands of scientists had a publication rate of approximately one paper every 5 days. With the advent of generative artificial intelligence (generative AI), such rate is likely to increase (2). In a world astonished by the potential of ChatGPT (a chatbot powered by GPTs, Generative Pre-trained Transformer), released by OpenAI in late 2022 (3), it seems effortless to generate high-quality written text using focused prompts. Since its launch, this innovative and user-friendly tool has rapidly reached a large portion of the public, and its Large Language Models (LLMs) applications, previously confined to a limited number of specialists, are now accessible to everyone.

In medical scientific writing, generative AI has shown its potential. The ease with which it can help produce and improve an article is astonishing (4). The applications are many: from programming codes to support for statistical analysis to editing manuscripts (5) to being a valuable tool for generating evidence synthesis and meta-analyses, which, with the integration of AI technologies, can be completed in a matter of days instead of months (6). This astounding use makes one look forward to enhancing research capabilities and better synthesizing applicable information across professional fields.

As we enhance the ability and speed by which we can synthesize and write about research, an important question arises: will this affect the number and quality of publications and the ability to utilize them effectively?

This paper explores the implications of the increasing use of generative AI in medical writing, focusing on its effects on health professionals and the potential for an AI-driven infodemic. By addressing existing gaps in the literature, the study provides a critical analysis of how these technological developments challenge the quality, equity, and reliability of medical knowledge production while proposing solutions to mitigate potential risks.

## Current status of literature production

In recent years, there has been a significant increase in the number of scientific articles submitted to academic journals. Clark (7) pointed out that journal submissions have increased by more than 60%, with nearly a quarter of a million submissions in the first wave of the COVID-19 pandemic. For example, BMJ journal submissions rose by almost 20% in 2020 compared to the previous year. While the presence of a large-scale emergency, such as a pandemic, is expected to increase the demand and production for scientific writing, this trend cannot be attributed to the pandemic effect alone. As Esther Landhuis (8) noted, the production of scientific papers increased by 8–9% annually over the past few decades. The growing need to accommodate this large number of publications has led to the creation of new academic journals, often charging publication fees. The number of scholarly journals has risen significantly, from just under 35,000 in 2010 to over 46,000 in 2020 (9), including an increase in the number of medical journals adopting an open-access policy (10). As a consequence, this may lead to an overload of the peer review process and represents a challenge for researchers who are competent in a specific field but lack the time to complete the reviews (10–12). Under typical circumstances, it can take several months to complete a peer review process (13), but considering the growing amount of submissions, reviewers may improperly use generative AI to perform peer review on their behalf (14). This practice may undermine the quality of the scientific publication process and introduce errors into the published literature, thus contributing to the mechanisms of an AI-driven infodemic of scientific papers filled with unchecked and unreliable information. In addition, non-peer reviewed knowledge dissemination channels, such as pre-prints portals, have found ample space further impacting the information ecosystem. When amplified by social media and digital platforms, this overflow of preliminary or low-quality evidence may contribute to the spread of health misinformation, with potential downstream effects on clinical practice and population health (15). Although researchers argue that even non-peer reviewed articles may include important findings and influence scientific progress in a positive manner (16), it is reasonable to assume that practitioners may feel overwhelmed when deciding which source to trust when dealing with such an infodemic of scientific production.

The concept of infodemic has been widely described in scientific literature (17) as “the rapid spread of large amounts of sometimes conflicting or inaccurate information that can impede the ability of individuals, communities, and authorities to protect health and effectively respond in a crisis” (18). This phenomenon inevitably affects the quality of the information produced and the extent to which it can impact practitioners and policymakers’ decision-making, due to an inevitable mix of reliable and unreliable information that can be further expanded by generative AI, a phenomenon named AI-driven infodemic (19).

## The publish or perish culture

This surge of publications is also linked to the mounting pressure to produce and disseminate research findings at any cost. This pressure, often referred to as the “publish or perish” culture, underscores the imperative for academic researchers to meet bibliometric benchmarks, secure research funding, and attain more prestigious academic positions (20). Evidence suggests that such pressure can shift the focus from producing high-quality, impactful research to maximizing publication numbers, sometimes leading to fragmented or redundant studies, selective reporting, and other questionable research practices (21, 22). Generative AI can amplify this trend by accelerating the production of manuscripts—whether based on primary/secondary data or commentaries—driven more by the need to publish than by genuine improvements in methodological rigor or to fill a real gap in knowledge (2). This may further increase low-quality publications, complicating the identification of reliable evidence. In such context, LLMs become catalysts of the “publish or perish” culture, becoming tools that support the researchers’ needs for visibility.

## The risk of healthcare decisions driven by unreliable scientific production

LLMs’ vast amount of information might compromise the peer review system and the validity of scientific outputs. On this premise, inaccurate content could be inserted by mistake or intentionally for fraudulent purposes. A notable example is a proof-of-concept study showing the capacity of ChatGPT to generate fabricated but highly convincing medical articles, complete with references and data, which could easily have deceived readers and even expert reviewers (23). Another striking case occurred when an AI-generated article was published in a predatory journal under the false authorship of a well-known academic, complete with a valid DOI, despite the fact that the researcher had never submitted or written the work (24). Such incidents highlight how AI can be misused to create fraudulent outputs, which, once disseminated, may acquire unwarranted credibility and influence. For example, unreliable information could be used to favor one pharmaceutical product over another, steering scientific debate or prescription trends. In the era of LLMs, the speed and scale at which incorrect claims about a pharmaceutical product or medical procedure could be generated and disseminated—potentially perceived as credible—greatly increases the risk of misguided healthcare decisions and the spread of mis-disinformation, potentially amplified by an AI-fueled and unverified infodemic (19). Recent evidence shows that digital misinformation during the pandemic directly influenced harmful behaviors, ranging from the inappropriate use of hydroxychloroquine to reduced vaccine uptake. This demonstrates the concrete impact of health misinformation on population-level outcomes (25, 26).

## Who would be most impacted?

As with all types of threats, there are vulnerable groups that will be most at risk from the effect of an infodemic. In low-income countries, health professionals may lack the resources to manage an infodemic of scientific literature (27). For example, they may find it more difficult to publish in higher-quality journals, increasing their exposure to less reputable or predatory journals (28, 29). Furthermore, the shift toward open-access publishing has also created inequalities for authors in economically disadvantaged areas, who are often unable to afford article-processing charges (APCs) (30). Private initiatives such as “Research4Life” are focused on finding solutions to this issue, providing researchers in low-income countries with free access to paid journals (31). However, it is questionable whether these programs can fill the inequality gap in a context lacking medical-scientific training and the ability to critically appraise the scientific literature (32). The advent of AI-driven infodemics threatens to widen the gap between health professionals with varying degrees of susceptibility to this overload of information, exacerbating the already existing disparity in clinical and research skills across countries and undermining equity in producing and using scientific findings. Another concern is the potential interference of generative AI in this field of knowledge reproduction, including academic and non-academic teaching, which ultimately impacts trainees’ critical thinking abilities and independent problem-solving skills. The use of AI for content generation could significantly impact professionals and trainees, as an optimally packaged scientific output might discourage further analysis and investigation of the subject matter being researched and studied. One foreseeable consequence is deskilling, leaving professionals unable to navigate entire publication processes independently. This could undermine healthcare quality and evidence-based medicine in an already weak professional environment (33), as the inability to critically appraise and produce scientific evidence may lead to the uncritical adoption of outdated, biased, or low-quality sources, ultimately resulting in inappropriate clinical decisions and reduced patient safety.

## The future is now: it is time to act!

To date, numerous attempts have been made to replicate original articles using non-human authors. Many readers and scientists have already recognized typical AI writing patterns in early reports (34). At the same time, AI-generated articles and images have been retracted (35). As a result, several policies are emerging to regulate the use of AI in academia, with publishing group policies requiring disclosure of AI use and full author accountability. However, the impact of these policies on applicability and compliance remains unknown. Despite developing detectors that recognize AI-generated language (36), there are no reliable standards for accurately detecting AI-written text. Furthermore, whether a tool can detect text produced by current LLMs, let alone more sophisticated future versions, is still debated. This situation underscores the need for clear guidance in navigating the vast and complex “mare magnum” of information.

In the context of this AI-driven infodemic, researchers and practitioners must identify reliable sources of information to ensure the high quality of their work. As demonstrated by the use of hydroxychloroquine during the COVID-19 pandemic, the impact of non-peer-reviewed or low-quality literature may lead to the utilization of inappropriate therapies (37). Given this premise, it is evident that an increase in the number of publications can lead to an imbalance between the supply of information and the practitioners’ ability to extract useful content (38). This phenomenon raises the question of how to select which articles to use. One potential solution is to rely on systematic reviews and meta-analyses, which consolidate the evidence from multiple sources into one article. However, even these types of studies may be subject to the same pitfalls in an infodemic context. Various reviews may exist on the same topic, with subtle differences between them. In addition, reviews may be inconclusive due to the poor quality of the articles included (39).

## Conclusion

There is a balance to be struck between the use of AI to rapidly produce scientific literature and the ability to select, critically appraise, and utilize the production of literature by clinicians, public health practitioners, and policymakers. From this perspective, it may be helpful to consider strategies that can be implemented. Regulation of the use of AI for scientific writing can enable its use to be standardized and good practices to be addressed on a common front, providing standards of action that can improve its results and enable its full potential to be exploited. However, regulation alone cannot be the only way to manage such a disruptive phenomenon. This is why it may be useful to adopt a more holistic approach, recalibrating training methods in a global sense. This recalibration should encompass not only the technical aspect of the quality of information but also the ethical considerations that come with the production of information and how health professionals and researchers are trained. The ethical use of AI in scientific literature can be ensured only when health professionals possess the skills to critically assess both its potential risks and its substantial benefits. While numerous educational programs and scholarly discussions on the ethics and applications of AI in healthcare are already available (40), a considerable proportion of professionals remain outside the reach of such training initiatives. Therefore, educational opportunities should be designed to reach all professionals, regardless of seniority or institutional affiliation, and should be made freely accessible. Ensuring open-access and cost-free training is crucial to avoid inequalities in digital literacy and to guarantee widespread adoption of ethical and responsible practices. The absence of adequate digital literacy and ethical guidance among these individuals increases the likelihood of inappropriate or harmful use of AI tools. In parallel, academic institutions are encouraged to promote transparent ethical guidelines on the acceptable use of generative AI in manuscript drafting, ensuring that disclosure of AI support becomes a standardized practice. Journals should establish clear editorial policies to verify compliance and provide examples of best practices. At the same time, researchers must be trained to integrate AI tools responsibly, using them as support rather than substitution for scientific reasoning and authorship accountability. In contrast, there is a pressing need for tools that assess the quality of a publication, the data it contains, the reproducibility of the research itself, and the impact it has on clinical practice or policymaking to support quality, soundness, and ethical principles rather than mere productivity.
